# Utility of High-Sensitivity Modified Glasgow Prognostic Score in Cancer Prognosis: A Systemic Review and Meta-Analysis

**DOI:** 10.3390/ijms24021318

**Published:** 2023-01-10

**Authors:** Tsung-Hsien Wu, Yao-Te Tsai, Kuan-Yin Chen, Wing-Keen Yap, Chih-Wei Luan

**Affiliations:** 1 Institute of Health Policy and Management, National Taiwan University, Taipei 100, Taiwan; 2 Legal Affair Department, New Taipei City Department, New Taipei City 220242, Taiwan; 3 College of Medicine, Chang Gung University, Taoyuan 333, Taiwan; 4 Department of Otorhinolaryngology-Head and Neck Surgery, Chang Gung Memorial Hospital, Chiayi 613, Taiwan; 5 School of Dentistry, National Yang Ming University, Taipei 11221, Taiwan; 6 Department of Radiation Oncology, Proton and Radiation Therapy Center, Chang Gung Memorial Hospital-Linkou Medical Center, Taoyuan 333423, Taiwan; 7 Department of Otorhinolaryngology-Head and Neck Surgery, LO-Sheng Hospital Ministry of Health and Welfare-Home, New Taipei City 242, Taiwan; 8 General Education Center, Lunghwa University of Science and Technology, Taoyuan 33306, Taiwan; 9 Graduate Institute of Clinical Medical Sciences, College of Medicine, Chang Gung University, Taoyuan 333, Taiwan

**Keywords:** high-sensitivity modified Glasgow Prognostic Score, cancer, survival outcome, meta-analysis

## Abstract

The suitability of the high-sensitivity modified Glasgow Prognostic Score (HS-mGPS) in cancer patients remains unknown. We performed a systematic database search from 1 January 2010 to 30 September 2022, in accordance with the Preferred Reporting Items for Systematic Reviews and Meta-Analyses guidelines. Selected studies reported the HS-mGPS and survival outcomes in cancer patients. The association between the HS-mGPS and survival outcomes was evaluated using a random-effects model and expressed as pooled hazard ratios (HRs) with 95% CIs. This meta-analysis evaluated 17 studies with a total of 5828 cancer patients. A higher HS-mGPS was found to be associated with an adverse OS (HR = 2.17; 95% CI: 1.80–2.60), DSS (HR = 3.81; 95% CI: 2.03–7.17), and DFS (HR = 1.96; 95% CI: 1.48–2.58; all *p* ≤ 0.001). The prognostic value of the HS-mGPS for the OS trended in a consistent direction after subgrouping and sensitivity analysis. In conclusion, the HS-mGPS serves as a valid prognostic biomarker for cancer patients, with a high HS-mGPS associated with adverse survival outcomes.

## 1. Introduction

Cancer constitutes one of the leading causes of mortality worldwide and is responsible for nearly 10 million deaths per year; one in five people develop cancer during their lifetime, with an estimated 19.3 million new cases detected in 2020 [1]. Despite advancements in early detection, surgical modalities, chemotherapy regimens, radiotherapy, immune therapies, and multidisciplinary treatments, cancer patients continue to confront unsatisfactory prognoses, with 1 in 10 dying from the disease [1,2]. Current cancer prognoses and treatment strategies are based on the staging system, which focuses solely on tumor characteristics. Tumor genetic composition and affiliated carcinogenic infections, such as human papillomavirus (HPV), Epstein–Barr virus, and hepatitis B and C viruses, have been widely scrutinized [3,4]. In addition, increasing evidence suggests that the nutritional and immunological conditions of the patient are associated with tumor progression and development [5,6,7]. Therefore, the identification of key biomarkers that help predict survival outcomes and aid in treatment optimization is crucial for cancer patients. 

Roxburgh and McMillan validated the role of systemic inflammatory response in the survival predictions for patients with primary operable cancer in 2010 [8]. Previous investigations have indicated that circulating inflammatory cells and immune mediators play a critical role in the tumor microenvironment and influence tumorigenesis, metastasis, and cancer progression [9,10]. The Glasgow Prognostic Score (GPS), which combines C-reactive protein and albumin levels that reflects the systemic inflammation status and the nutrition status of the patient, respectively, has been reported to be a reliable tool for assessing the status of cancer cachexia [11,12]. The GPS and modified GPS (mGPS) have been examined and validated for having independent prognostic value in cancer patients, across various types of cancers, in more than 60 studies including over 30,000 patients [13]. However, the high-sensitivity mGPS (HS-mGPS), which is based on a more stringent serum CRP level (3 mg/L) cutoff, has been reported to act as a better prognostic indicator for various malignancies than the conventional mGPS [14,15,16]. In general, the HS-mGPS was defined as follows: Patients with both hypoalbuminemia (<35 g/L) and increased CRP levels (>3 mg/L), with one of these variables, and with none of these variables were assigned scores of 2, 1, and 0, respectively. This study investigated whether the HS-mGPS provides an accurate prognosis of survival outcomes in cancer patients.

## 2. Materials and Methods

### 2.1. Database Search Strategy

This systematic review and meta-analysis followed the Preferred Reporting Items for Systematic Reviews and Meta-Analyses guidelines [17]. This meta-analysis was registered with PROSPERO (no. CRD42022376554). Two authors performed structured and independent database searches of PubMed, the Cochrane Library, and Embase from 1 January 2010 to 30 September 2022. The following MeSH terms and free text words were used: “high-sensitivity modified Glasgow prognostic scores”, “HS-mGPS”, “cancer”, “carcinoma”, “metastasis”, “tumor”, “tumour”, and “neoplasms.” Details on the search strategy for each database are provided in Supplementary Table S1. Initially, the two authors (Luan CW and Wu TH) responsible for conducting the database search screened the identified titles and abstracts for eligibility, and a third author (Yap WK) was consulted to resolve any disagreements. We reviewed the reference lists of the retrieved articles to identify additional relevant papers. No restrictions on language or publication year were applied to minimize publication bias. The researchers also searched ClinicalTrials.gov to identify relevant ongoing trials. Institutional Review Board approval was waived because no individual data were used.

### 2.2. Eligibility Criteria

The study inclusion criteria were as follows: (1) enrolling patients with all cancer types; (2) investigating the association of the HS-mGPS with survival outcomes; and (3) providing hazard ratios (HRs) with 95% CIs or sufficient information to enable their calculation. The following were excluded: (1) reviews, meta-analyses, conference abstracts, case reports, letters, or commentaries; (2) studies lacking data on survival outcomes; and (3) studies enrolling patients who had participated in a previously published trial. Two authors (Luan CW and Wu TH) independently screened all study titles and abstracts, excluded irrelevant studies, and reviewed the full texts of the remaining studies. A third author (Yap WK) was consulted in cases when the aforementioned authors could not reach a consensus.

### 2.3. Data Extraction

**Figure 1:**
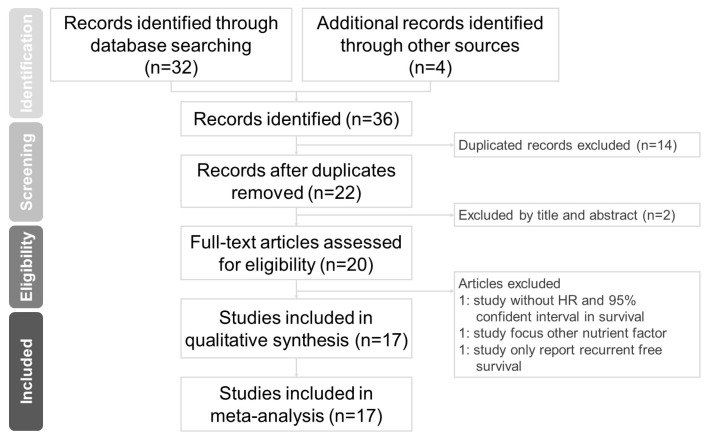
Flowchart of study selection.

Two independent authors (Luan CW and Wu TH) extracted the following data from the evaluated studies: (1) study characteristics (author name, study region, sample size, study duration, median follow-up time, and year of publication); (2) participant characteristics (cancer type, cancer stage, staging system, and treatment method); (3) selected cutoff values of the HS-mGPS for survival analysis; and (4) HRs with 95% CIs for overall survival (OS), disease-free survival (DFS), and disease-specific survival (DSS). If a study provided the HRs with 95% CIs of an HS-mGPS of 1 versus 0 and an HS-mGPS of 2 versus 0 as two groups, we calculated the data as two datasets. If a study presented the results in both multivariable and univariable analysis, the adjusted HRs of the multivariable analysis were used for analysis [18]. Formal analysis was conducted from 30 September to 20 November 2022. The flowchart of study selection is summarized in Figure 1.

### 2.4. Quality Assessment

We used the Newcastle–Ottawa Quality Assessment Scale (NOS), which consists of eight main elements for study quality evaluation [19], when assessing the methodological quality of the evaluated studies. Studies with a high methodological quality NOS score of ≥7 out of 9 were enrolled in this meta-analysis. Any scoring disagreements were resolved through discussion. 

### 2.5. Statistical Analysis 

A random-effects model was applied to incorporate survival outcomes in the current meta-analysis due to the expectation of heterogeneity within the included studies [20]. Comprehensive Meta-Analysis Version 3 (Biostat, Englewood, NJ, USA) was used to perform all statistical analyses. Statistical significance was indicated by a two-tailed *p* value of <0.05. We used pooled HRs with 95% CIs to quantify the primary study outcomes (OS, DSS, and DFS), and the results of the pooled data were adopted to investigate the associations between the HS-mGPS and patient prognoses. An *I*^2^ test and Cochran’s Q test were also conducted to assess heterogeneity between studies. In Cochran’s Q test, statistical significance was indicated at *p* < 0.1. *I*^2^ values of ≤24.9%, 25% to 49.9%, 50% to 74%, and ≥75% indicated no, low, moderate, and high heterogeneity, respectively [18]. We performed a subgroup analysis in cases of significant heterogeneity and used a sensitivity analysis to evaluate the robustness of the pooled results. We investigated potential publication bias using funnel plots in >10 studies [18].

## 3. Results 

### 3.1. Literature Search

The literature search and selection process are summarized in Figure 1. A total of 36 articles were identified through structured database searches. After the removal of 14 duplicate articles and 2 nonrelevant articles, 20 full-text articles were assessed for eligibility. Among the remaining studies, 3 studies that failed to meet the inclusion criteria were removed. The present meta-analysis included a total of 17 studies.

### 3.2. Study Characteristics 

The general characteristics of the included studies are summarized in Table 1. All were retrospective studies from Asian countries (Japan, Taiwan, and China) that were written in English and published between 2014 and 2022. The sample sizes ranged from 70 to 1625 (median: 163). Among them, 13 studies [7,14,15,16,21,22,23,24,25,26,27,28,29] (4651 cases) used multivariable analysis and 4 studies [30,31,32,33] (1177 cases) used univariable analysis in survival outcome measurement. This meta-analysis analyzed multiple cancer types, including gastrointestinal cancer (3 gastric cancers [16,30,31], 1 hepatocellular carcinoma [28], 1 esophageal cancer [14], 1 gallbladder cancer [32], and 1 colorectal cancer [29]), head and neck cancer (1 head and neck cancer [22], 1 oropharyngeal cancer [26], 1 hypopharyngeal cancer [27], and 1 oral cancer [7]), soft tissue sarcoma (3 soft tissue sarcoma [21,23,33] and 1 neuroblastoma [24]), non-small-cell lung cancer [15] (n = 1), and prostate cancer [25] (n = 1). A total of 12 studies reported cancer stage according to the TNM staging system, while 6 studies reported stage according to other staging systems, including the French Federation of Cancer Centers Sarcoma Group grading system, the Barcelona Clinic Liver Cancer staging system, the Japanese Gastric Cancer Treatment Guidelines, and the International Neuroblastoma Staging System. All studies focused on the association between the pretreatment HS-mGPS and survival outcomes. The reported adjusted HRs with 95% CIs for survival outcomes, including OS, PFS, DSS, and DFS, were directly extracted from the included studies. The NOS scores of the studies ranged from 7 to 9, with the detailed assessment process presented in Supplementary Table S2.

### 3.3. Prognostic Effect of the HS-mGPS for OS 

**Figure 2:**
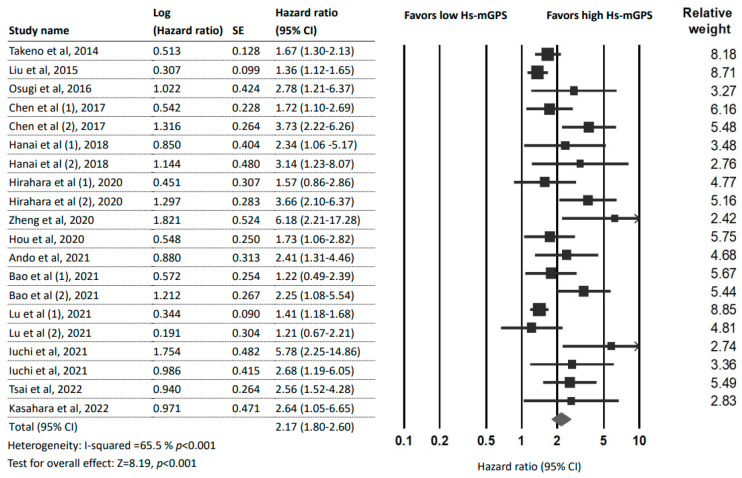
Forest plot indicating the pooled HRs to assess the influence of the HS-mGPS on OS. Abbreviations: HR, hazard ratio; CI, confidence interval; HS-mGPS, high-sensitivity modified Glasgow Prognostic Score [7,14,15,16,22,23,24,25,26,27,28,29,30,31,32].

Fifteen studies [7,14,15,16,22,23,24,25,26,27,28,29,30,31,32] (20 datasets and 5545 patients) reported an association between the HS-mGPS and OS. The pooled analysis detected a significant association between a high HS-mGPS and poor OS in cancer patients (HR = 2.17; 95% CI: 1.80–2.60; *p* < 0.001) with moderate heterogeneity (*I*^2^ = 65.5%; *p_H_* < 0.001; Figure 2). 

Subgroup analyses were conducted to further examine the consistency of the prognostic role of the HS-mGPS for OS. We divided the 20 datasets groups based on tumor site, research region, sample size, HS-mGPS cutoff value, and analysis method. The results are presented in Table 2. The subgroup analyses indicated a significant relationship between a high HS-mGPS and poor OS in patients with different cancer types, including gastrointestinal cancer (HR = 1.90; 95% CI: 1.53–2.35), head and neck cancer (HR = 2.88; 95% CI: 2.07–4.01), soft tissue sarcoma (HR = 2.10; 95% CI: 1.41–3.40), lung cancer (HR = 2.78; 95% CI: 1.21–6.38), and prostate cancer (HR = 2.41; 95% CI: 1.31–4.45). The high heterogeneity of tumors in gastrointestinal cancer (*I*^2^ = 70.14%) and soft tissue sarcoma (*I*^2^ = 84.0%) but not in head and neck cancer (*I*^2^ = 0%) suggested that the prognostic value of the HS-mGPS may vary by tumor type. We observed a significant and constant association between the HS-mGPS and OS in the subgroups based on research region, sample size, HS-mGPS cutoff value, and analysis method; these results support the consistency of our findings and indicate that a high HS-mGPS likely predicts poor OS. Mild-to-moderate heterogeneity was detected across the included studies (*I*^2^ = 36.15–78.90%), with the exception of the cutoff value = 1 subgroup (*I*^2^ = 0%). The varied heterogeneity across subgroups may provide additional insight into the possible causes of heterogeneity observed in the pooled results.

### 3.4. Prognostic Effect of the HS-mGPS on DFS

**Figure 3:**
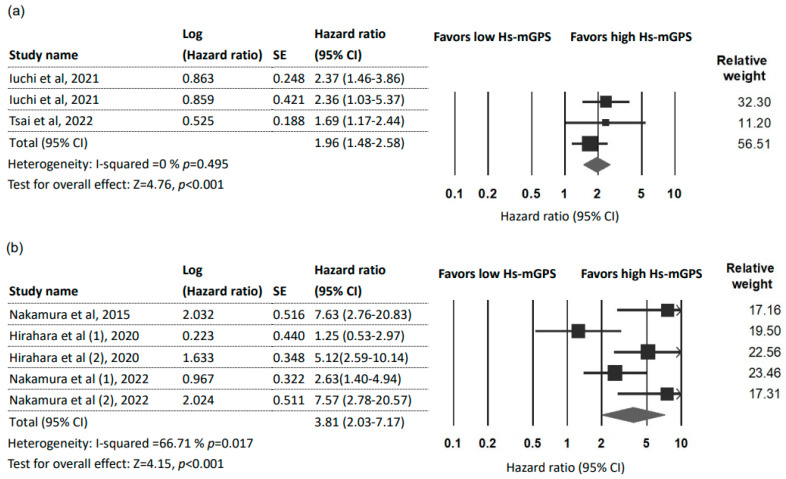
Forest plot indicating the pooled HRs to assess the influence of the HS-mGPS. (**a**) The influence of the HS-mGPS on DFS; (**b**) The influence of the HS-mGPS on DSS. Abbreviations: HR, hazard ratio; CI, confidence interval; HS-mGPS, high-sensitivity modified Glasgow Prognostic Score [7,21,26,27,31,33].

Three studies [7,26,27] with 524 patients reported a prognostic role of the HS-mGPS for DFS. The pooled results indicated the HS-mGPS to be an independent factor that can predict adverse DFS (HR = 1.96; 95% CI: 1.48–2.58; *p* < 0.001) with no heterogeneity (*I*^2^ = 0%; *p_H_* = 0.459; Figure 3a). Furthermore, these three studies all focused on cancers of the head and neck region (oral, oropharyngeal, and hypopharyngeal). Due to a limited number of studies, subgroup and sensitivity analyses were not performed. Begg’s test and Egger’s test did not detect significant publication bias in the studies.

### 3.5. Prognostic Effect of the HS-mGPS on DSS

Three studies [21,31,33] with five datasets (717 patients) were used to analyze the association between the HS-mGPS and DSS. The pooled results indicated that a high HS-mGPS exerted a significant prognostic effect on DSS in cancer patients (HR = 3.81; 95% CI: 2.03–7.17; *p* < 0.001; Figure 3b) with moderate heterogeneity (*I*^2^ = 66.71%; *p_H_* = 0.017). No significant publication bias was detected by Begg’s test and Egger’s test. Subgroup and sensitivity analyses were not performed because of the limited number of available studies.

### 3.6. Publication Bias and Sensitivity Analysis

**Figure 4:**
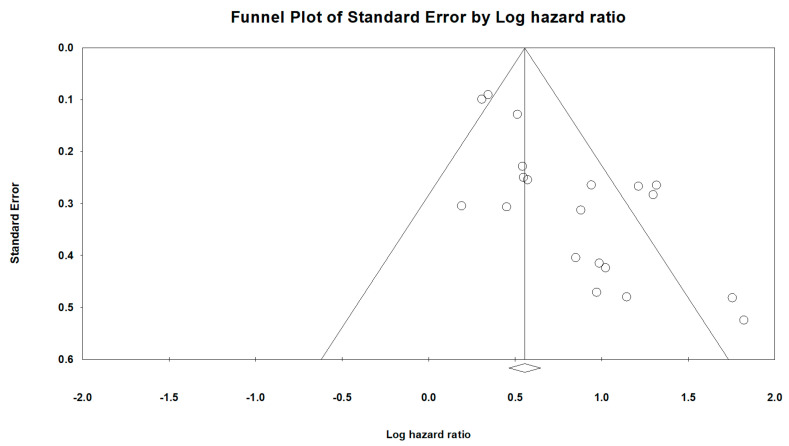
Funnel plots for the included studies in this meta-analysis. Each point represents a separate study. The two sloping lines indicate the 95% CI.

A visual inspection of the funnel plots that were designed to estimate the publication bias for OS of the HS-mGPS (Figure 4) revealed obvious asymmetry, indicative of an insufficient number of studies, which were characterized by small HRs and small sample sizes. Significant publication bias was also confirmed by Begg’s test (*p* < 0.001) and Egger’s test (*p* < 0.001). Therefore, we used the trim-and-fill method to evaluate the influence of publication bias on the pooled results. The results from the trim-and-fill calculation indicated a pooled HR of 1.63 (95% CI: 1.34–2.00; *p* < 0.001) for OS prediction after adding the missing studies. The smaller adjusted HR did not alter the direction or significance of the results, a finding that supports the validity of this meta-analysis. In addition, sensitivity analyses were conducted through the individual omission of studies from the pooled analysis to assess the stability of the results with regard to OS (Supplementary Table S3). The removal of any individual study did not significantly alter the influence of the HS-mGPS on OS, reinforcing the reliability of our results.

## 4. Discussion

This meta-analysis combined data from 15 studies (5545 patients) and determined a statistically significant association between the HS-mGPS and OS of cancer patients; statistical significance was maintained in sensitivity analyses. The background conditions of patients varied but did not exert a statistically significant effect on the direction of the associational between the HS-mGPS and OS. The subgroup analysis results indicated that the prognostic value of the HS-mGPS for OS maintained significance for cancer type, sample size, cutoff value of the HS-mGPS, research region, and analysis method (Table 2). As we know that the tumor stage may affect the prognostic value, we carefully reviewed all the included studies and the detail covariates of multivariable analysis (Supplementary Table S4). In the multivariable analysis group (only the adjusted HRs were included in the analysis), there were 17 datasets with 4512 patients and the pooled result showed HR = 2.24 (1.83–2.75), *p* < 0.001. Of note, “stage” was adjusted in the multivariate models in all of the studies included in the subgroup analysis. In the subgroup analyses stratified by cutoff value of the HS-mGPS, the HR of the group HS-mGPS = 2 was greater than that of the group HS-mGPS = 1 and ≥1 (HR = 2.82, 1.51, and 2.20, respectively), suggesting an association between high HS-mGPS and poor OS outcome. Similarly, a high HS-mGPS in cancer patients served as an unfavorable prognostic factor for DFS and DSS. This study is the first systematic review or meta-analysis to demonstrate the prognostic role of the HS-mGPS with respect to survival outcomes.

Growing evidence has revealed that ratios based on components of the white cell count are positively associated with tumor malignancy, including the neutrophil-to-lymphocyte ratio, the platelet-to-lymphocyte ratio, the monocyte-to-lymphocyte ratio, and the Systemic Immune–Inflammation Index [34,35]. Furthermore, systemic inflammatory response biomarkers such as the CRP-to-albumin ratio and the Prognostic Nutrition Index are also known to have prognostic value in cancer [6,36]. However, disputed cutoff values for these markers render them difficult to use in the survival predictions of different populations. Additionally, studies have noted the independent prognostic value of the GPS/mGPS, a novel inflammatory index, for patients with various cancers [13,37]. The HS-mGPS, a new but well-established scoring system introduced by Proctor and McMillan, modified the serum CRP threshold from 10 mg/L (conventional mGPS) to 3 mg/L to enhance the index’s prognostic capacity [38]. The HS-mGPS is a biomarker produced by the integration of two laboratory indices (CRP and albumin) and independently serves as a convenient and cost-effective biomarker in routine clinical practice. Some research has suggested that the prognostic value of the HS-mGPS surpasses that of NLR, PLR, and conventional mGPS for certain cancers [7,14,15,16,23]. Unlike white blood-cell-based marker testing, the HS-mGPS is not included in the standard preoperative work-up. The incorporation of the HS-mGPS into the preoperative work-up of cancer patients may help establish evidence in favor of this score’s clinical utility and expand its recognition and acceptance.

How the HS-mGPS is relevant to adverse prognoses in cancer patients remains undetermined. An elevated HS-mGPS is indicative of a patient with hypoalbuminemia, a high CRP level, or both. CRP acts as an acute phase plasma protein in response to inflammation or infection; it is regulated by proinflammatory cytokine stimulation such as tumor necrosis factor-alpha, interleukin-1 (IL-1), and IL-6 [39,40]. In addition, CRP suppresses the immune response and accelerates cancer migration and tumor microenvironment formation [41]. The optimal cutoff point of CRP is significant. Cohort studies on cancer and other diseases have agreed with the hypothesis of McMillan and Proctor, the doctors who first validated mGPS and suggested that raising the CRP level by >3 mg/L would achieve better predictive value [38,42,43,44]. Further, hypoalbuminemia caused by malnutrition and cancer cachexia indicates nutritional deficiency, sarcopenia, and poor patient performance, which may negatively influence cancer prognoses [45]. Cancer-related inflammation can impair albumin synthesis through changes in cytokine production that increase microvascular permeability [46]. Thus, a high HS-mGPS combined with a high CRP and hypoalbuminemia may indicate strong systemic inflammation and poor nutritional status and be associated with poor survival outcomes in cancer patients.

The staging system, an essential element of the cancer treatment strategy, nevertheless excludes individual factors. Clinicians explain to patients that individual factors account for the varying prognoses of patients who are at the same stage but with different prognoses. With this fact in mind, a simple, readily available, and reliable score system that can generate valuable prognostic data may serve as helpful treatment information for clinicians. The development of personalized treatment regimens is of vital importance; therefore, many studies have discovered better prognosis tools by focusing on a combination of a staging system with the immunological and nutritional status of the patient. The results of this meta-analysis summarized the existing evidence of the effectiveness of the HS-mGPS in survival prognosis and endorsed its prognostic value for cancer patients. However, several studies that we analyzed which compared the mGPS and the HS-mGPS indicated a higher prognostic value for the HS-mGPS [14,15,16]. Additional prospective studies are required to further investigate the prognostic effect of the two scores in cancer patients.

This meta-analysis has several limitations. First, all included studies were retrospective and displayed considerable methodological diversity. The inclusion of various cancer types, cancer stages, and treatment strategies may have contributed to the high statistical heterogeneity observed in our meta-analysis. However, the sensitivity test and subgroup analysis displayed no statistically significant effect on the direction of the association between the HS-mGPS and survival outcomes. Second, there were no published reports from American or European countries, with all included studies examining Asian populations. The global application of our findings remains undetermined. Moreover, the meta-analysis had statistically significant publication bias; however, a trim-and-fill analysis did not alter the direction or significance of the results. Finally, the HRs obtained in the studies were calculated with a combination of multivariable analysis (13 studies) and univariable analysis (4 studies), which may have influenced the reliability of the findings. Therefore, we advise others to interpret and use our results cautiously, and we encourage further prospective studies to reinforce our findings.

## 5. Conclusions

The current study suggested that high HS-mGPS has an adverse effect on survival in cancer patients. This meta-analysis reviewed the existing evidence of the usefulness of the HS-mGPS as a biomarker and confirmed its reliability for predicting cancer prognosis. Future large-scale, prospective clinical trials are required to further validate our study results in the global population.

## Figures and Tables

**Table 1:** The characteristics of included studies.

Omitted Study	Year	Country	Study Period	Study Design	Survival Analysis	Sample Size	Cancer Type	Cancer Stage	Treatment	Outcome	Median Follow-Up (Months)	NOS Score
Takeno et al. [16]	2014	Japan	1995–2006	retrospective	Multivariable	494	Gastric cancer	I–IV * (7th UICC)	OP ± adjuvant therapy	OS	NR	8
Nakamura et al. [21]	2015	Japan	2001–2012	retrospective	Multivariable	139	Soft tissue sarcoma	Grade 1–3 (FNCLCC)	OP/RT/CT	DSS	60	8
Liu et al. [30]	2015	China	2005–2010	retrospective	Univariable	455	Gastric cancer	I–III	D2 gastrectomy with R0 resection	OS	25	7
Osugi et al. [15]	2016	Japan	2005–2009	retrospective	Multivariable	327	Non-small cell lung cancer	I–III * (7th UICC)	OP	OS	65	9
Chen et al. [14]	2017	China	2011–2014	retrospective	Multivariable	163	Esophageal cancer	II–Iva (6th AJCC)	CCRT	OS	NR	7
Hanai et al. [22]	2018	Japan	2012–2013	retrospective	Multivariable	129	Head and neck cancer	I–IV (7th UICC)	OP/CCRT	OS	43.6	8
Hirahara et al. [31]	2020	Japan	2010–2017	retrospective	Univariable	434	Gastric cancer	I–IV * (4th JGCTG)	OP	OS/DSS	NR	9
Zheng et al. [24]	2020	China	2008–2016	retrospective	Multivariable	70	Neuroblastoma	1–4 (INSS)	OP ± adjuvant therapy/CT	OS	53.1	9
Hou et al. [23]	2020	China	2000–2016	retrospective	Multivariable	454	Soft tissue sarcoma	I–III (AJCC), Grade 1–3 (FNCLCC)	OP/RT/CT	OS	94.8	9
Ando et al. [25]	2021	Japan	2005–2019	retrospective	Multivariable	131	Castration-resistant prostate cancer	IV	ADT plus docetaxel	OS/PFS	21.1	8
Bao et al. [32]	2021	China	2010–2017	retrospective	Multivariable	144	Gallbladder cancer	I–IV * (8th AJCC)	OP	OS	NR	8
Lu et al. [28]	2021	China	2006–2014	retrospective	Multivariable	1625	Hepatocellular carcinoma	ABC (BCLC-C)	TACE	OS	NR	7
Iuchi et al. [26]	2021	Japan	2009–2020	retrospective	Multivariable	106	Oropharyngeal cancer	I–IV * (8th AJCC)	OP/CCRT	OS/DFS	42	8
Iuchi et al. [27]	2021	Japan	2007–2019	retrospective	Multivariable	115	Hypopharyngeal cancer	II–IV * (8th AJCC)	OP/CCRT	OS/DFS	62	8
Nakamura et al. [33]	2022	Japan	2002–2018	retrospective	Univariable	144	Soft tissue sarcoma	Grade 1–3 (FNCLCC)	OP	DSS	76	9
Tsai et al. [7]	2022	Taiwan	2008–2017	retrospective	Multivariable	303	Oral squamous cell carcinoma	I–IV * (8th AJCC)	OP ± adjuvant therapy	OS/DFS	40.9	9
Kasahara et al. [29]	2022	Japan	2000–2015	retrospective	Multivariable	595	Colorectal cancer	II–IV *	OP ± adjuvant therapy	OS	NR	7

* excluded metastasis. Abbreviations: AJCC, American Joint Committee on Cancer; UICC, Union for International Cancer Control tumor-node-metastasis; FNCLCC, French Federation of Cancer Centers Sarcoma Group grading system; BCLC-C, Barcelona Clinic Liver Cancer-Stage C; JGCTG, Japanese Gastric Cancer Treatment Guidelines; INSS, International Neuroblastoma Staging System; OP, operation; RT, radiotherapy; CT, chemotherapy; ADT, androgen depletion therapy; NR, not reported.

**Table 2:** Main results of stratified analyses for the impact of HS-mGPS on overall survival.

Subgroup	No. of Datasets (Patients)	HR (95% CI)	*p* Value	Heterogeneity Test	
				*I*^2^ %	*p* Value
Total	20 (5545)	2.17 (1.80–2.60)	<0.001	65.5	<0.01
Tumor site					
Gastrointestinal cancer	11 (4073)	1.90 (1.53–2.35)	<0.001	70.14	<0.01
Gastric cancer	4 (1383)	1.79 (1.28–2.49)	=0.001	73.41	0.01
Esophageal cancer	2 (163)	2.50 (1.17–5.34)	0.018	79.64	0.03
Hepatocellular carcinoma	2 (1625)	1.39 (1.18–1.65)	<0.001	0	0.63
Gallbladder cancer	2 (144)	2.43 (1.30–4.55)	0.006	66.86	0.08
Colorectal cancer	1 (595)	2.64 (1.05–6.64)	0.039		
Head and neck cancer	5 (653)	2.88 (2.07–4.01)	<0.001	0	0.62
Oropharyngeal cancer	1 (106)	5.78 (2.25–14.85)	<0.001		
Hypopharyngeal cancer	1 (115)	2.68 (1.19–6.04)	0.017		
Oral cancer	1 (303)	2.56 (1.53–4.30)	<0.001		
Mixed	2 (129)	2.64(1.44–4.85)	0.002	0	0.64
Soft tissue sarcoma	2 (524)	2.19 (1.41–3.40)	<0.001	79.19	0.03
Lung cancer	1 (327)	2.78 (1.21–6.38)	0.016		
Prostate cancer	1 (131)	2.41 (1.31–4.45)	0.005		
Region					
China	9 (2911)	1.92 (1.48–2.48)	<0.001	73.74	<0.01
Japan	10 (2331)	2.43 (1.88–3.15)	<0.001	37.21	0.11
Taiwan	1 (301)	2.56 (1.53–4.30)	<0.001		
Sample size					
<165	10 (858)	2.75 (2.12–3.56)	<0.001	36.15	0.12
≥165	10 (4687)	1.74 (1.44–2.10)	<0.001	55.08	0.02
Cutoff value of HS-mGPS					
2	6 (2822)	2.82 (1.95–4.07)	<0.001	50.77	0.07
1	5 (2495)	1.51 (1.30–1.75)	<0.001	0	0.64
≥1	9 (2723)	2.20 (1.66–2.92)	<0.001	65.76	0.01
Analysis method					
Multivariable	17 (4656)	2.24 (1.83–2.75)	<0.001	60.83	<0.01
Univariable	3 (889)	1.92(1.06–3.50)	=0.032	81.67	<0.01

Abbreviations: CI, confidence interval; HR, hazard ratio; HS-mGPS, high-sensitivity modified Glasgow Prognostic Score; No., number.

## Data Availability

The data presented in this study are available on request from the corresponding author.

## Supplementary Materials

The following supporting information can be downloaded at: https://www.mdpi.com/article/10.3390/ijms24021318/s1.
