# Supplementary material for: Utility of High-Sensitivity Modified Glasgow Prognostic Score in Cancer Prognosis: A Systemic Review and Meta-Analysis
Source: Int J Mol Sci. 2023 Jan 10;24(2):1318. doi: 10.3390/ijms24021318 (PMC9866297; doi:10.3390/ijms24021318)
Supplement: Supplementary file 1 [file ijms-24-01318-s001.zip › Table S1. search strategy.pdf]

Supplementary Table S1 | Detailed search strategies.

CENTRAL (via Cochrane Register of Studies)	PubMed	EMBASE (Ovid)
#1 MeSH descriptor: [Neoplasms] explode all trees<br>#2(cancer* or carcinoma* or neoplasm* or tumor* or tumour* or metastas*):ti,ab,kw<br>#3 #1 OR #2<br>#4 (modified Glasgow Prognostic Score OR mGPS):ti,ab,kw<br>#5 (high-Sensitivity Modified Glasgow Prognostic Scores OR HS-mGPS):ti,ab,kw<br>#6 #4 OR #5<br>#7 #3 AND #6	#1 "neoplasms"[MeSH Terms]<br>#2 "cancer*"[Title/Abstract] OR "carcinoma*"[Title/Abstract] OR "neoplasm*"[Title/Abstract] OR "tumor*"[Title/Abstract] OR "tumour*"[Title/Abstract] OR "metastasis*"[Title/Abstract]<br>#3 #1 OR #2<br>#4 "modified glasgow prognostic score"[Title/Abstract] AND "mGPS"[Title/Abstract]<br>#5 "high-Sensitivity Modified Glasgow Prognostic Scores" [Title/Abstract]) AND "hs-mGPS" [Title/Abstract])<br>#6 #4 OR #5<br>#7 #3 AND #6	#1 neoplasm<br>#2 'cancer*':ab,ti OR 'carcinoma*':ab,ti OR 'neoplasm*':ab,ti OR 'tumor*':ab,ti OR 'tumour*':ab,ti OR 'metastas*':ab,ti<br>#3 #1 OR #2<br>#4 'modified glasgow prognostic score':ti,ab,kw OR mgps:ti,ab,kw<br>#5'high-sensitivity modified glasgow prognostic scores':ti,ab,kw OR 'hs mgps':ti,ab,kw<br>#6 #4 OR #5<br>#7 #3 AND #6
