# Supplementary material for: Utility of High-Sensitivity Modified Glasgow Prognostic Score in Cancer Prognosis: A Systemic Review and Meta-Analysis
Source: Int J Mol Sci. 2023 Jan 10;24(2):1318. doi: 10.3390/ijms24021318 (PMC9866297; doi:10.3390/ijms24021318)
Supplement: Supplementary file 1 [file ijms-24-01318-s001.zip › Table S3. Sensitivity analysis of the association between mGPS and overall survival.pdf]

Supplementary Table S3. Sensitivity analysis of the association between Hs-mGPS and overall survival.

Study omitted	HR (95% CI)	*p*	I^2	P_H
Takeno et al, 2014 [16]	2.24(1.83-2.75)	< 0.001	67.22%	<0.001
Liu et al, 2015 [30]	2.27(1.86-2.76)	<0.001	61.63%	<0.001
Osugi et al, 2016 [15]	2.15(1.78-2.59)	< 0.001	66.55%	<0.001
Chen et al (1), 2017 [14]	2.21(1.82-2.70)	< 0.001	67.29%	<0.001
Chen et al (2), 2017 [14]	2.07(1.73-2.48)	< 0.001	61.28%	<0.001
Hanai et al (1), 2018 [22]	2.16(1.79-2.62)	< 0.001	66.97%	<0.001
Hanai et al (2), 2018 [22]	2.14(1.78-2.58)	< 0.001	66.36%	<0.001
Hirahara et al (1), 2020 [31]	2.21(1.82-2.68)	<0.001	67.22%	<0.001
Hirahara et al (2), 2020 [31]	2.09(1.74-2.50)	< 0.001	62.48%	<0.001
Zheng et al, 2020 [24]	2.10(1.75-2.51)	< 0.001	63.39%	<0.001
Hou et al, 2020 [23]	2.20(1.81-2.68)	<0.001	67.29%	<0.001
Ando et al, 2021 [25]	2.16(1.78-2.61)	< 0.001	66.63%	<0.001
Bao et al (1), 2021 [32]	2.20(1.81-2.67)	< 0.001	67.29%	<0.001
Bao et al (2), 2021[32]	2.10(1.74-2.52)	< 0.001	63.10%	<0.001
Lu et al (1), 2021 [28]	2.27(1.86-2.77)	< 0.001	62.08%	<0.001
Lu et al (2), 2021 [28]	2.23(1.85-2.70)	<0.001	66.40%	<0.001
Iuchi et al, 2021 [26]	2.09(1.75-2.50)	< 0.001	63.10%	<0.001
Iuchi et al, 2021 [27]	2.15(1.78-2.60)	< 0.001	66.63%	<0.001
Tsai et al, 2022 [7]	2.15(1.77-2.60)	<0.001	65.94%	<0.001
Kasahara et al, 2022 [29]	2.16(1.78-2.60)	< 0.001	66.82%	<0.001
