# Supplementary material for: Utility of High-Sensitivity Modified Glasgow Prognostic Score in Cancer Prognosis: A Systemic Review and Meta-Analysis
Source: Int J Mol Sci. 2023 Jan 10;24(2):1318. doi: 10.3390/ijms24021318 (PMC9866297; doi:10.3390/ijms24021318)
Supplement: Supplementary file 1 [file ijms-24-01318-s001.zip › Table S4. Multivariate models and covariates used in the studies.pdf]

Supplementary Table S4. Multivariate models and covariates used in the studies.

Study name	Multivariate models	Covariates for adjust
Takeno et al, 2014 [16]	Cox proportional hazards model	Depth of tumor invasion, Node metastasis, Distant metastasis
Osugi et al, 2016 [15]	Cox proportional hazards model	Age, p-T status, p-N status, Blood vessel invasion, Lymphatic vessel invasion
Chen et al, 2017 [14]	Cox regression model	ECOG PS, T stage, N stage, Clinical stage, Tumour length, BMI (Kg/m2), Treatment modality, Tumour response
Hanai et al, 2018 [22]	Cox proportional hazards models	ECOG PS, TMN stage,
Zheng et al, 2020 [24]	Cox proportional hazards model	Age, INSS stage, Tumor size, Metastasis
Hou et al, 2020 [23]	Cox proportional hazard model	Tumour size, Tumour depth, Tumour grade, AJCC stage
Ando et al, 2021 [25]	Cox proportional hazard models	Bone metastasis, PSA ≥ 27.0 ng/mL, Alkaline phosphatase ≥ 293 IU/L, Testosterone ≥ 13.0 ng/dL
Bao et al, 2021 [32]	Cox proportional hazards model	Nerve invasion, Differentiations, Liver invasion, T stage, N stage, TNM stage, Serum CEA level, Serum CA 19-9 level
Lu et al, 2021 [28]	Cox proportional hazards models	ALBI grade, AFP level, tumor size, Macroscopic vascular invasion, Extrahepatic metastasis, BCLC stage
Iuchi et al, 2021 [26]	Cox proportional hazards models	ECOG PS, Tumor stage, Nodal stage, AJCC stage
Iuchi et al, 2021 [27]	Cox proportional hazards models	Tumor stage, Nodal stage, AJCC stage
Tsai et al, 2022 [7]	Cox proportional hazard model	Cell differentiation, Overall stage, pT classification, pN classification, ENE, DOI ≥ 10 mm, Adjuvant therapy
Kasahara et al, 2022 [29]	Cox regression model	BMI, T stage, N stage, CEA, CA199,
