# Supplementary material for: Utility of High-Sensitivity Modified Glasgow Prognostic Score in Cancer Prognosis: A Systemic Review and Meta-Analysis
Source: Int J Mol Sci. 2023 Jan 10;24(2):1318. doi: 10.3390/ijms24021318 (PMC9866297; doi:10.3390/ijms24021318)
Supplement: Supplementary file 1 [file Table S2. NOS.pdf › Table S2. NOS.pdf]

Supplementary Table S2. Indices of methodological quality of the studies according to the Newcastle–Ottawa Quality Assessment Scale for cohort studies

	**Selection**				**Comparability**	**Outcome**			
**Studies**	Representativeness of the exposed cohort	Selection of the non exposed cohort	Ascertainment of exposure	Demonstration that outcome of interest was not present at start of study	Comparability of cohorts on the basis of the design or analysis	Assessment of outcome	Was follow-up long enough for outcomes to occur	Adequacy of follow up of cohorts	Total scores (stars)
Takeno et al, (2014) [16]	*	*	*	*	*	*	*	*	8
Nakamura et al, (2015) [21]	*	*	*	*	*	*	*	*	8
Liu et al, (2015) [30]	*	*	*	*	*	*	-	*	7
Osugi et al, (2016) [15]	*	*	*	*	**	*	*	*	9
Chen el al, (2017) [14]	*	*	*	*	*	*	-	*	7
Hanai et al, (2018) [22]	*	*	*	*	*	*	*	*	8
Hirahara et al, (2020) [31]	*	*	*	*	**	*	*	*	9
Zheng et al, (2020) [24]	*	*	*	*	**	*	*	*	9
Hou et al, (2020) [23]	*	*	*	*	**	*	*	*	9
Ando et al, (2021) [25]	*	*	*	*	**	*	-	*	8
Bao et al, (2021) [32]	*	*	*	*	**	*	-	*	8
Lu et al, (2021) [28]	*	*	*	*	*	*	-	*	7
Iuchi et al, (2021) [26]	*	*	*	*	*	*	*	*	8
Iuchi et al, (2021) [27]	*	*	*	*	*	*	*	*	8
Nakamura et al, (2022) [33]	*	*	*	*	**	*	*	*	9
Tsai et al, (2022) [7]	*	*	*	*	**	*	*	*	9
Kasahara et al, (2022) [29]	*	*	*	*	*	*	-	*	7
